# Microarray analysis reveals marked intestinal microbiota aberrancy in infants having eczema compared to healthy children in at-risk for atopic disease

**DOI:** 10.1186/1471-2180-13-12

**Published:** 2013-01-23

**Authors:** Lotta Nylund, Reetta Satokari, Janne Nikkilä, Mirjana Rajilić-Stojanović, Marko Kalliomäki, Erika Isolauri, Seppo Salminen, Willem M de Vos

**Affiliations:** 1 Functional Foods Forum, University of Turku, Turku, FI-20014, Finland; 2 Department of Veterinary Biosciences and Department of Bacteriology and Immunology, University of Helsinki, Helsinki, Finland; 3 Finnish Red Cross Blood Service, Helsinki, Finland; 4 Laboratory of Microbiology, Wageningen University, Wageningen, The Netherlands; 5 Department for Biotechnology and Biochemical Engineering, Faculty of Technology and Metallurgy, University of Belgrade, Belgrade, Serbia; 6 Department of Pediatrics, University of Turku and Turku University Hospital, Turku, Finland

**Keywords:** Infant, Intestinal microbiota, Microbiota diversity, Phylogenetic microarray, Eczema

## Abstract

**Background:**

Deviations in composition and diversity of intestinal microbiota in infancy have been associated with both the development and recurrence of atopic eczema. Thus, we decided to use a deep and global microarray-based method to characterize the diversity and temporal changes of the intestinal microbiota in infancy and to define specific bacterial signatures associated with eczema. Faecal microbiota at 6 and 18 months of age were analysed from 34 infants (15 with eczema and 19 healthy controls) selected from a prospective follow-up study based on the availability of faecal samples. The infants were originally randomized to receive either *Lactobacillus rhamnosus* GG or placebo.

**Results:**

Children with eczema harboured a more diverse total microbiota than control subjects as assessed by the Simpson’s reciprocal diversity index of the microarray profiles. Composition of the microbiota did not differ between study groups at age of 6 months, but was significantly different at age of 18 months as assessed by MCPP (p=0.01). At this age healthy children harboured 3 -fold greater amount of members of the Bacteroidetes (p=0.01). Microbiota of children suffering from eczema had increased abundance of the *Clostridium* clusters IV and XIVa, which are typically abundant in adults. Probiotic *Lactobacillus rhamnosus* GG supplementation in early infancy was observed to have minor long-term effects on the microbiota composition.

**Conclusion:**

A diverse and adult-type microbiota in early childhood is associated with eczema and it may contribute to the perpetuation of eczema.

## Background

Major microbial colonization of the gastrointestinal tract starts at delivery when an infant comes into contact with the environment. The composition of developing microbiota is affected by factors such as mode of delivery [1-3], dietary pattern [4,5] and administration of probiotics or antibiotics [6,7]. The early colonization events and the commensal intestinal microbiota shape the immune system and potentially affect the development of variety of diseases [8].

Previous studies have shown associations between the composition of intestinal microbiota and atopic diseases. Most of these have addressed the microbiota composition preceding the development of atopic disease, while microbiota aberrancies in infants already suffering from eczema have obtained less attention. Reduced diversity at early life (i.e. at 1 week, 1 month or 4 months of age) has been associated with an increased risk of developing atopic disease [9-12]. The results on specific bacterial species or groups that would either increase or decrease the risk of developing allergy are still conflicting [13-15].

Few studies have observed microbiota alterations in allergic children (i.e. after the onset of allergy) with also conflicting results [16-19]. For example, faecal bifidobacterial counts have been reported to be both decreased [17,18] or similar [16] as compared to healthy children. Similarly, microbiota diversity in allergic children was found to be decreased in one study [19] but not in another [16]. Notably, the studies were conducted by using traditional cultivation-based techniques or molecular techniques that target a sub-set of the intestinal microbiota. Finally, the administration of specific probiotic bacteria during pregnancy and/or during the first months of life has been shown to reduce the risk of atopy, especially atopic eczema [20-23]. However, some studies have failed to find any connection between the microbiota composition and development of atopic eczema [24] or to confirm the role of probiotics in prevention of atopic diseases [25,26].

A variety of high-throughput methods based on 16S ribosomal RNA (rRNA) gene sequence analysis have been established to analyse the intestinal microbiota in a culture-independent way, including next -generation sequencing analysis and phylogenetic microarrays [27]. The high-density phylogenetic microarray HITChip consists of 3699 unique 16S rRNA gene targeting oligonucleotide probes that selectively recognise microbes at different taxonomic levels [28]. This and other microarrays have shown to be instrumental for the comprehensive and high-resolution analysis of the microbiota composition from microbial species (or phylotypes) to phylum-like level [28-30].

The objective of this study was to characterize the diversity and temporal changes of intestinal microbiota in early childhood and to identify specific bacterial groups associated with eczema. By using the HITChip microarray and strategic qPCR analysis of early life fecal samples, we detected specific differences in microbiota composition between healthy children and those with eczema.

## Methods

### Study design, subjects and faecal samples

Subjects of this study represent a sub-population from a prospective follow-up trial at Turku University Central Hospital, Finland, which has been described in detail previously [20]. Briefly, the inclusion criterion for the children was that they had a high risk of atopic diseases, i.e. they had at least one close relative (mother, father and/or sibling) with atopic eczema, allergic rhinitis or asthma. Further inclusion criteria for present study were vaginal delivery after full-term pregnancy (≥ 37 weeks), normal birth weight (≥ 2500 g) and the availability of faecal samples taken at the ages of 6 and/or 18 months. Finally, all infants were exclusively or partially breast-fed for at least four months. Based on these criteria, 34 children from the original study population (n= 132) [20] were included in this study. The basic characteristics of the study subjects are shown in Additional file 1.

Mothers were randomized to receive capsules containing either placebo or 1 × 10^10^ colony-forming units of *Lactobacillus rhamnosus* GG (ATCC 53103) daily for 2–4 weeks before expected delivery. The intervention continued 6 months postnatally. The capsule contents were consumed by mothers during the exclusive breastfeeding, otherwise infants received the agents. The occurrence of eczema was diagnosed by the age of 2 years by typical skin lesions found in children and chronic relapsing course. This last criterion was fulfilled if the child had had eczema for 1 month or longer at the 24-month study visit and on at least one previous visit (at ages 3, 6, 12, 18 months). Eczema was considered atopic if it was associated with positive skin prick test(s) at 6 and/or 24 -month study visit. None of the study subjects included in present study suffered from asthma or allergic rhinitis. Also, all the infants were normal weight at the age of 6 and 18 months of age.

The study protocol was approved by the Ethics Committee of the Hospital District of Southwest Finland and subjects were enrolled in the study after written informed consent was obtained.

### Faecal samples and DNA extraction

The faecal samples were taken from children at age of 6 and 18 months. The samples were aliquoted and frozen immediately after collection, and stored in −80°C. DNA was extracted from faecal samples using the repeated bead-beating method as described previously [31,32].

### 16S rRNA gene microarray analysis

The composition of total microbiota was assessed by using the phylogenetic Human Intestinal Tract chip (HITChip) as described previously [28,33], except for the amplification step, where 25 cycles of end-point PCR were used. Microarray analysis of all samples were performed in at least two independent hybridizations until satisfactory reproducibility was achieved (>96%). This study reports results of more than 150 independent microarray hybridizations.

The HITChip is a custom-made Agilent microarray (Agilent Technologies, Palo Alto, CA, USA) designed to comprehensively cover the diversity of the human intestinal microbiota. The array contains 3699 unique oligonucleotide probes targeting the V1 and V6 hypervariable regions of the 16S rRNA gene and covering over 1100 intestinal bacterial phylotypes. The HITChip allows the analysis at three phylogenetic levels: phylum-like level (level 1), genus-like level (level 2) and phylotype level (species-like, level 3). The details of the HITChip have previously been described, including its validation for phylogenetic fingerprinting and quantification [28].

### Microarray data extraction and microbiota diversity assessment

Data were extracted from microarray images using the Agilent Feature Extraction software, version 9.5.1 (http://www.agilent.com). Normalization of microarray data was performed as described earlier [28,34]. Further data processing was performed by using a custom designed relational database running under the MySQL database management system (http://www.mysql.com) using R-based scripts [28].

### Quantitative PCR

Quantitative PCR (qPCR) analysis of *Bifidobacterium* genus and species was carried out in an Applied Biosystems 7300 Fast Real-Time PCR System in a 96-well format and by using SYBR Green chemistry (SYBR Green PCR Master Mix, Applied Biosystems, USA). The primers and their specificities are presented in Additional file 2. The PCR reactions and thermocycling conditions were as reported earlier [35,36]. Standards for qPCR were prepared as described in Nermes et al. [37]. Samples were analysed in duplicate in at least two independent runs.

### Statistical and data analyses

Statistical analysis of both qPCR and HITChip data was carried out with log-transformed data. In qPCR data, non-detected values were imputed with the half of the theoretical detection limit. For HITChip data, linear models with factors for treatment, health status, time point and breast-feeding with subsequent ANOVA and contrast tests were used to determine the statistical differences between groups. In microarray data, cut-off values for positive responding probes were calculated as described before [28]. In HITChip data the analysed values were summary values on phylum-like and genus-like level, obtained by summing the intensities from all the probes assigned to the respective phylum-like or genus-like phylogetic groups. Totally 19 phylum-like and 78 genus-like level groups reached the detection threshold and were thus used in statistical analysis. The data is presented as mean with standard deviation values. Redundancy analysis (RDA) was performed by using the multivariate statistical analysis package Canoco [38]. RDA plot shows bacterial groups principally contributing to the difference between the groups of subjects. The significance of separation in RDA was assessed by Monte Carlo Permutation Procedure (MCPP [39]).

The diversity of the microbial community assessed by HITChip was expressed as Simpson’s reciprocal index of diversity (1/D) as described before [28,40].

## Results

### Temporal development of microbiota

**Figure 1:**
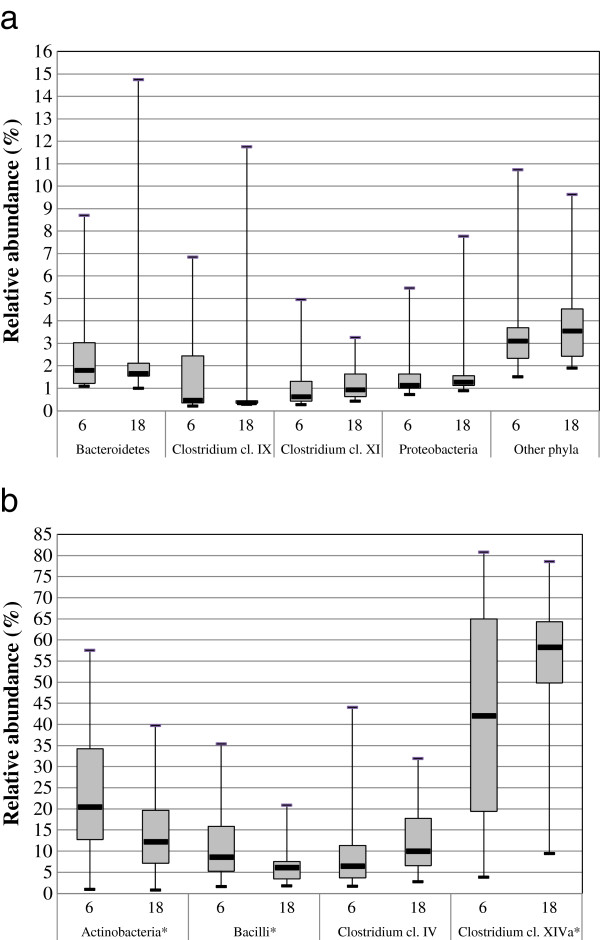
**Relative contribution of phylum-like bacterial groups to the total HITChip signals of infants at 6 and 18 months of age.** Groups contributing for at least 1% (**a**) and at least 5% (**b**) to the profiles are presented in the legend. The box extends from 25^th^ percentile to 75^th^ percentile, with a line at the median; the whiskers extent to the highest and lowest values. * Statistically significant change (p < 0.05).

The faecal microbiota of 34 children at age of 6 and 18 months was analysed using the HITChip phylogenetic microarray. The diversity of total microbiota increased significantly with age, as the Simpson’s the reciprocal diversity index has changed from 78 ± 24 to 111 ± 27 at age of 6 and 18 months, respectively (p < .001). At the phylum-like level, significant changes in the relative abundances of major bacterial groups were detected (Figure 1). The most prominent decline in abundance was observed for Actinobacteria that contributed 24.2% and 14.1% to the total signal at 6 and 18 months of age, respectively (p= 0.01). Signal intensities for Actinobacteria were almost entirely obtained from bifidobacteria (22.9% of the total microbiota at 6 months and 12.6% at 18 months, p= 0.01). This finding was consistent with quantitative PCR analysis, where total bifidobacteria counts decreased significantly with age (p= 0.03, Additional file 3). At the species level, the amounts of *B. longum/infantis* group, *B. breve*, *B. bifidum, B. catenulatum* group and *B. adolescentis* decreased over time as assessed by qPCR. In addition to Actinobacteria, the relative abundance of Bacilli decreased with age (from 11.8% to 7.1%, p= 0.03). All genus-like groups belonging to Bacilli decreased, most of which not significantly as individual groups, but the sum effect at the phylum-like level was significant (Figure 1). An opposite trend was observed for the bacterial groups belonging to the major *Clostridium* clusters, especially members of the *Clostridium* cluster XIVa which relative abundance increased with age from 42.0% to 55.6% (p= 0.02).Similar was observed for *Ruminococcus bromii* et rel. group from *Clostridium* cluster IV that increased from 0.13% to 0.34% (p=0.01). In total, 21 genus-like phylogenetic groups changed significantly with age, (Table 1), which further highlights the extensive compositional changes that the microbiota is undergoing during this period of life.

**Table 1:** Genus-like phylogenetic groups changing statistically significantly from 6 to 18 months of age as assessed by HITChip analysis

**Phylum/order**	**Genus-like phylogenetic group**	**Mean relative abundances (SD)**		
		**6 months**	**18 months**	**p-value**
Actinobacteria	*Bifidobacterium*	22.86 (15.92)	12.61 (9.51)	0.01
Bacilli	*Lactobacillus plantarum* et rel.	3.64 (5.41)	0.32 (0.49)	0.006
*Clostridium* cluster IV	*Ruminococcus bromii* et rel.	0.13 (0.25)	0.35 (0.37)	0.01
*Clostridium* cluster IX	*Phascolarctobacterium faecium* et rel.	0.06 (0.01)	0.07 (0.01)	0.001
*Clostridium* cluster XIVa	*Butyrivibrio crossotus* et rel.	0.65 (0.43)	1.03 (0.63)	0.01
	*Clostridium symbiosum* et rel.	3.45 (2.17)	4.87 (1.97)	0.018
	*Lachnobacillus bovis* et rel.	0.27 (0.21)	0.62 (0.60)	0.004
*Clostridium* cluster XVIII	*Coprobacillus catenaformis* et rel.	0.06 (0.01)	0.11 (0.07)	0.0002
Fusobacteria	*Fusobacteria*	0.07 (0.02)	0.09 (0.01)	0.001
Proteobacteria	*Proteus* et rel.	0.07 (0.02)	0.09 (0.02)	0.002
	*Sutterella wadsworthia* et rel.	0.08 (0.02)	0.10 (0.01)	0.003
Uncultured Mollicutes	Uncultured Mollicutes	0.12 (0.03)	0.14 (0.02)	0.002

Genus-like groups with a p-value less than 0.01 are presented in the table.

### Analysis of the intestinal microbiota composition in relation to the health status

**Figure 2:**
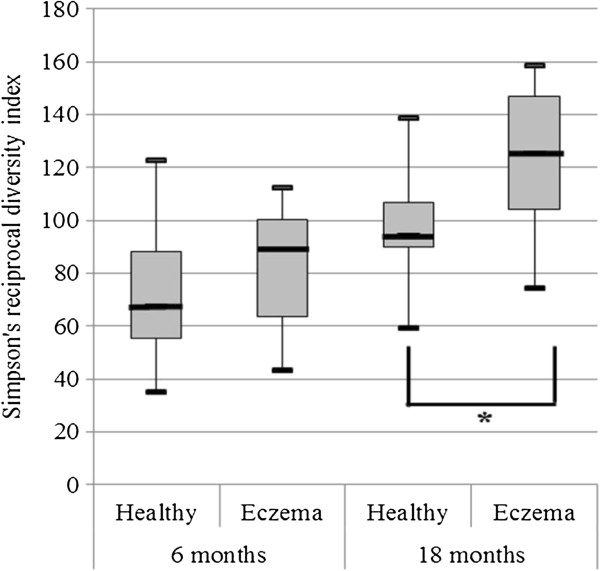
**Simpson’s reciprocal index of diversity in healthy children and children with eczema.** The box extends from 25^th^ percentile to 75^th^ percentile, with a line at the median; the whiskers extent to the highest and lowest values. * Statistically significant difference, p=0.03.

**Figure 3:**
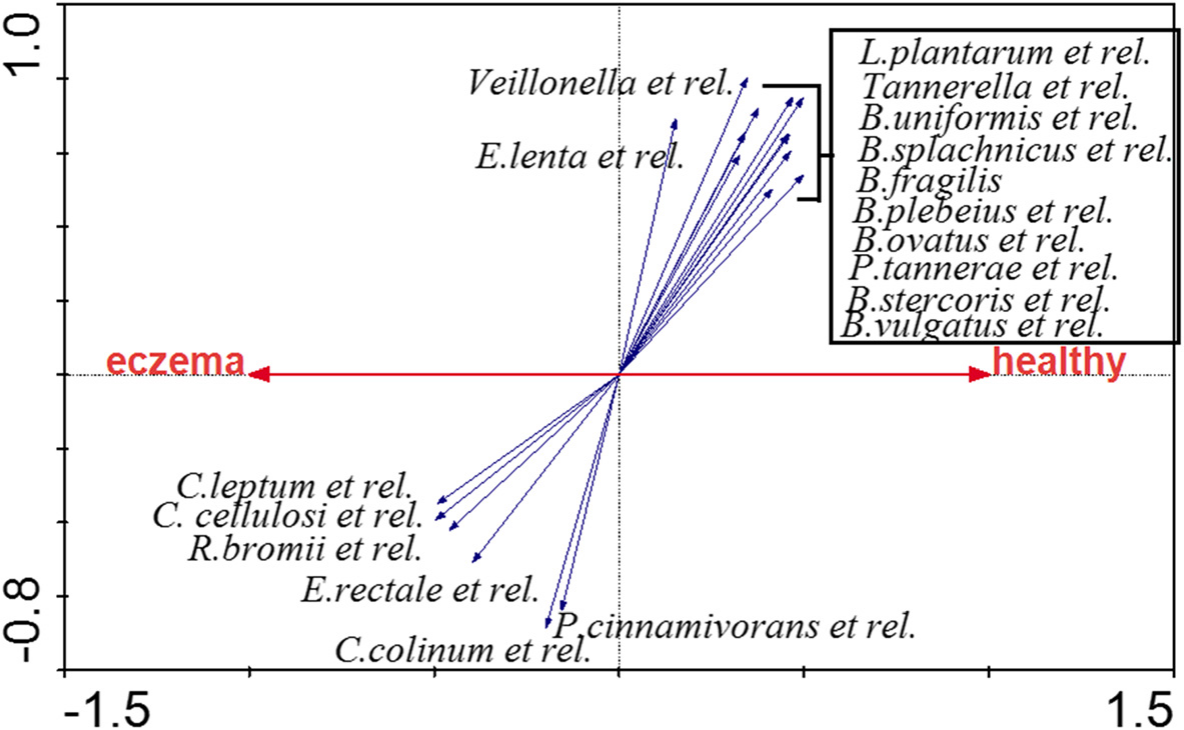
**RDA plot of the microbiota composition of healthy and eczematous children at 18 months of age.** Responding bacterial groups that contributed more than 35% of the variability of the samples are indicated by blue arrows. P-value obtained by Monte Carlo Permutation Procedure was 0.01. Abbreviations: B., *Bacteroides*, C., *Clostridium*, L., *Lactobacillus*, E., *Eggerthella*, Eub., *Eubacterium*, P., *Papillibacter*, R., *Ruminococcus*.

**Figure 4:**
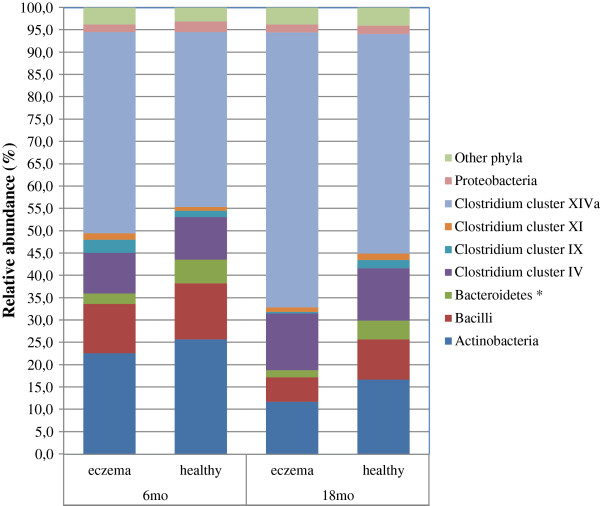
**Relative contribution of phylum-like bacterial groups to the total HITChip signals of healthy and eczematous infants at 6 and 18 months of age.** Groups contributing for at least 1% to the profiles are presented in the legend. * Statistically significant difference between healthy children and children with eczema at 18 months (p= 0.01).

When comparing the microbiota of the two groups of children at the age of 18 months, pronounced differences were observed both in the microbial composition and the diversity. Infants with eczema had a significantly more diverse total microbiota (p=0.03, Figure 2). Analysis at the species-like level showed that a large number of bacterial species have different abundance between healthy and eczematous infants, although the individual p-values are not particularly small (Additional file 4). The numerous, but mostly not significant, differences at the species-like level prompted us to look at the trends in microbiota differences at higher levels i.e. at the phylum-like and genus-like levels. Analysis at the phylum-like level showed that the most remarkable difference between the groups was observed in the Bacteroidetes, which were 3-fold more abundant in healthy children at the age of 18 months (p= 0.01, Table 2). The effect of Bacteroidetes is also clearly shown in the RDA plot (Figure 3), which reveals the bacterial groups principally contributing to the difference among the groups of subjects. The microbiota differences between the health groups shown in the RDA are significant as assessed by MCPP (p= 0.01) and a total of 9.1% of the variation within the dataset could be related to the health status of the infants. In contrast to the Bacteroidetes, specific bacterial groups from the most abundant groups of the Firmicutes phylum - *Clostridium* clusters IV and XIVa - were significantly more abundant in children with eczema (Table 2, Figure 4). In summary, the multiple differences in specific bacterial groups result in microbiota profiles that are significantly distinct between healthy and eczematous infants as assessed by MCPP (p=0.01, Figure 3).

**Table 2:** Statistically significant differences in microbiota of healthy and eczematous children

**Phylum-like level**	**Genus-like phylogenetic group**	Mean relative abundance* (SD)		
		**18 months**		**p-value**
		**Healthy**	**Eczema**	
Bacteroidetes		4.20 (4.21)	1.61 (0.36)	0.01
	*B. fragilis et rel*.	0.49 (0.74)	0.13 (0.03)	0.01
	*B. ovatus et rel*.	0.20 (0.23)	0.09 (0.02)	0.03
	*B. plebeius et rel*.	0.08 (0.03)	0.06 (0.01)	0.02
	*B. stercoris et rel*.	0.08 (0.03)	0.06 (0.01)	0.02
	*B. uniformis et rel*.	0.12 (0.21)	ND	< .001
	*B. vulgatus et rel*.	1.08 (1.80)	0.23 (0.15)	0.045
	*P. tannerae et rel*.	0.06 (0.04)	ND	0.03
*Clostridium* cluster IV	*C. leptum et rel*.	0.97 (1.36)	1.78 (1.19)	0.03
	*R. bromii et rel*.	0.25 (0.44)	0.44 (0.28)	0.03
	*C. cellulosi et rel*.	0.81 (0.78)	1.27 (0.65)	0.03
*Clostridium* cluster XIVa	*R. lactaris et rel*.	0.12 (0.16)	1.87 (2.83)	0.04
	*C. nexile et rel*.	1.65 (0.80)	2.05 (0.85)	0.02

* % of total HITChip signal

ND, below the detection level.

Similar microbiota changes were observed between children with eczema and healthy children when only subjects in the placebo group were analysed, although the differences between the groups did not reach statistical difference due to the small number of subjects (Additional file 5). No significant differences were observed for the bifidobacterial sub-community between the two groups of children using both HITChip and qPCR analyses (Additional file 6). The comprehensive list of phylum-like and genus-like level data and p-values obtained by statistical analyses are presented in Additional file 7 and Additional file 8, respectively.

Notably, an indication towards altered microbiota composition in children with eczema was already identified at 6 months, although the difference did not reach the level of statistical significance (MCPP, p=0.35). A higher abundance of the *Clostridium* cluster XIVa bacteria was observed in infants with eczema than healthy controls (mean relative abundances 45.1% and 39.1%, respectively, p= 0.50).

### *L. rhamnosus* GG supplementation in early infancy has minor long-term effects on the microbiota composition

When comparing the levels of HITChip signals between children from the placebo group and those who had received *L. rhamnosus* GG for their first 6 months of life, no statistically significant differences were observed at the age of 6 months. However, the supplementation with *L. rhamnosus* GG showed effects on three genus-like bacterial groups at the age of 18 months i.e. a year after the cessation of the probiotic supplementation. The children that had received *L. rhamnosus* GG had higher levels of the butyrate-producing groups *Anaerostipes caccae et rel* (LGG 2.89 ± 2.13% and placebo 1.18 ± 0.91% of the total microbiota, p=0.03) and *Eubacterium ventriosum et rel* (LGG 0.17 ± 0.11% and placebo 0.11 ± 0.07 of the total microbiota, p=0.04) than those of placebo group (Additional file 9). Moreover, the placebo group children had higher levels of *Clostridium difficile* et rel at 18 months of age as compared to the LGG group children (1.19 ± 0.85% and 0.78 ± 0.60%, respectively, p=0.047). The comprehensive list of phylum-like and genus-like level data and p-values obtained by statistical analyses are presented in Additional file 7 and Additional file 8, respectively.

The effect of the probiotic supplementation on the microbiota composition within the group of healthy children or the group of children with eczema was not addressed due to the small number of subjects.

## Discussion

We used a high-throughput phylogenetic microarray to reveal alterations in the gut microbiota composition throughout early childhood. The used microarray has been developed and validated for determining the microbiota diversity and evaluating the relative proportions of genus-like or higher (phylum-like) phylogenetic groups [28]. Moreover, it has been demonstrated that this microarray has a power equal to or higher than deep new generation sequencing [41] and it has previously been used to compare the microbiota diversity and composition in a variety of studies [33,42].

Despite the highly significant increase of microbiota diversity with age, the diversity indeces at 18 months of age are still relatively low (~110) when compared to the approximately two-fold higher indexes (150–200) commonly observed in healthy adults [32]. It has been suggested that by the age of 1 to 2 years the microbiota resembles that of an adult [29,43]. Our results show that microbiota succession continues at least until the age of 18 months and most likely even further, because the bacterial diversity has still not reached the diversity of an adult person. Thus, significant changes can be expected to occur in even after 18 months of age.

Concerning the microbiota composition at 6 months of age, our results are in agreement with earlier studies [5,29], except that we observed significant colonization by bifidobacteria in most of the children (mean relative abundances 22.9% at 6 months and 12.6% at 18 months of age, respectively) while in the study of Palmer et al. [29] bifidobacteria were not detected, possibly due to differences in DNA extraction, PCR primers, demographic and geographic origin, dietary patterns of the infants or other confounding factors. Primers used for PCR are often not so optimal for bifidobacteria than for other species and thus, high GC bacteria may perform less well in such PCRs. Further, in our previous studies we have shown that mechanical lysis of faecal bacteria is essential and improves the detection of especially Gram-positive bacteria including bifidobacteria [32,44]. In the Palmer et al. study [29], mechanical lysis by bead-beating was not applied, which may have hampered the detection of bifidobacteria. Thus, we consider that the most likely explanation for the different results concerning bifidobacteria in our and Palmer et al. [29] study is the different DNA extraction methods used.

When comparing healthy and eczematous children we found statistically significant differences in microbiota composition only at 18 months of age. The total microbiota of children with eczema was found to become significantly more diverse than the microbiota of children who remained healthy by 18 months of age. Interestingly, the total microbiota and particularly Firmicutes diversity was higher in the eczema group children, although the difference with the healthy subjects was not statistically significant. Abrahamsson et al. described the infants as having atopic eczema during the first two years of life (diagnostics were done at 6, 12 and 24 months of age), but the age at the onset of symptoms was not clarified [9]. However, it can be concluded from the Abrahamsson et al. [9] and our study, both taking advantage of the high resolution microbiota assessment techniques, that after 1 year of age the total microbiota diversity in children either developing or having eczema is comparable or even higher than that of healthy children. Secretory IgA has been suggested to play a role in shaping the microbiota composition and diversity. Some early studies showed an association between the low levels of secretory IgA and the risk of developing atopy [45,46] and could suggest that the low IgA levels permit establishment of a wider variety of bacteria and explain the higher bacterial diversity in children with eczema observed in this study. However, more recent studies have shown a higher concentration of secretory IgA in children with allergic sensitization during the first 2 years of life [47,48].

Another possible explanation for the increased bacterial diversity in children with eczema is the decreased levels or altered repertoire of antimicrobial peptides secreted into the gut lumen. These peptides, such as alpha- and beta-defensins, have at least two key roles at the mucosal interface: contributing to the host defense against enteric bacterial attachment and homeostatic control of the intestinal bacterial ecosystem [49,50]. Recently, decreased alpha-defensin levels and increased beta-defensin levels were associated with increased risk of developing atopy [51]. To our knowledge, the levels of faecal antimicrobial peptides in children already having eczema have not been studied. However, a few studies have highlighted the role of alpha-defensins in microbiota composition and intestinal health. For example, genetic mutations resulting in decreased alpha-defensin expression have been associated with the susceptibility and severity of inflammatory bowel disease in humans and decreased alpha-defensins may have an effect on the differences observed in microbiota composition between healthy and diseased subjects [52]. Interestingly, mice deficient in production of active alpha -defensins were shown to have a decrease in Bacteroidetes [50]. The reason for decreased Bacteroidetes levels in children with eczema in this study remains unaccountable, but alpha-defensins provide one possible explanation for our observation. Also other host-dependent factors, such as the amount of mucus secretion and differences in mucus glycosylation (e.g. FUT2 secretor status) may have an influence on the microbiota diversity and composition, as recently reviewed by Maynard et al. [53]. Clearly, the role of intestinal IgA levels, antimicrobial peptides and mucus secretion in shaping the gut microbiota in healthy and eczematous children warrants for further investigation.

Our results emphasize that the microbiota diversity in children with eczema should be further studied by using high-resolution techniques in order to define the favourable course of bacterial succession in early childhood and toddler age and to evaluate possible means to influence it.

It was observed that children with eczema harbour more bacteria belonging to the *Clostridium* cluster IV and *Clostridium* cluster XIVa. These bacteria are among the most abundant microbial groups detected in the healthy adult intestine [54]. Thus, prematurely occurring changes towards an adult-type microbiota were observed to take place in children with eczema. It has been suggested that resident bacteria may shape the hosts’ physiology, among others, by modulating the expression of genes involved in intestinal functions, such as postnatal intestinal maturation and the maintenance of mucosal barrier [55]. It may be speculated that an infant-type microbiota supports adequate gut barrier function and tolerance against food allergens in an immature gut. Infant-type microbiota may fortify the normal mucosal barrier function e.g. by affecting the maturation of the gut epithelium and immune functions in an optimal way and decrease the low-grade intestinal inflammation observable in subjects with eczema [53,56]. Maintenance of adequate mucosal barrier function may also play a role in the level of sensitisation to food-derived compounds [57,58]. The complex host-microbe interactions in the intestinal epithelium are only recently beginning to be understood [53,59].

Furthermore, we observed decreased relative abundances of bacteria belonging to Bacteroidetes in children with eczema. Previous studies have reported an association between decreased amounts *Bacteroides* spp. and the development of atopy and increased risk for atopic sensitization [9,60,61]. Bacteria belonging to the Bacteroidetes are among the first groups colonizing the gut [15,29] and they are typical intestinal habitants in healthy adults [62]. *Bacteroides* spp. are specialized in the breakdown of complex plant polysaccharides [63] and their abundance has been associated with increased short-chain fatty acid concentrations in the infant gut after introduction of first solid foods [64]. Furthermore, *B. fragilis* polysaccharide has been shown in mice model to direct the cellular and physical maturation of the developing immune system via its ability to direct the development of CD4+ T cells, thus inducing the differentiation of Th1 lineage and correction of the Th1/Th2 imbalance [65]. Together with our findings, these results suggest the significance of *Bacteroides* spp. in the development and maintenance of healthy infant gut and balanced mucosal immunity and necessitate the role of these bacteria to be considered in future studies.

When comparing healthy children with children with eczema we found statistically significant differences in microbiota composition only at 18 months, but not at 6 months of age. Breast-feeding is known as a major factor influencing the microbiota composition in infancy [4,5]. At 6 months of age, the majority of children included in this study were still nursed and breast-feeding is likely to have had a strong influence on their microbiota composition at that age. Thus, it seems that breast-feeding could have evened up the microbiota differences between the healthy and eczematous children and masked the eczema-associated changes, which came apparent and measurable at 18 months of age after the withdrawal of breast-milk.

While many studies addressed the impact of *L. rhamnosus* GG on health parameters, the short and long-term effect on the intestinal microbiota has only received limited attention. In the present intervention, the supplementation of *L. rhamnosus* GG continued until the age of 6 months. Interestingly, no significant effect on the microbiota composition was observed at the age of 6 months, but instead the supplementation of *L. rhamnosus* GG in early life was observed to a induce long-term effect and small but significant changes between the intervention groups were observed one year later at the age of 18 months. The observation that the *C. difficile* et rel. group bacteria were lower in the LGG groups as compared to placebo is of particular interest. Previously, *Clostridium difficile* colonization at the age of 1 month has been associated with a higher risk of a diagnosis of atopic dermatitis at the age of 2 years [66]. The higher *Anaerostipes caccae et rel* levels in the children that had received the *L. rhamnosus* GG supplementation is also a potentially beneficial effect, because *A. caccae* produces butyrate, which is an energy source for epithelial cells of colonic mucosa [67]. Bacteria belonging to the *Eubacterium ventriosum* et rel group that were higher in the children that received the probiotic supplementation, also have shown to produce butyrate but have been less investigated. In mice, however, it has been shown that *E. ventriosum* was reduced in colitic mice as compared to non-colitic animals [68]. To our knowledge this is the first high -throughput microbiota analysis study reporting the long-term effects of a probiotic strain on the microbiota composition in early life.

## Conclusions

In conclusion, using a comprehensive microbial analysis approach we observed children with eczema to harbour a more diverse total microbiota and detected specific shifts in bacterial groups in different phylogenetic levels. The results indicate that aberrancies in microbiota composition are associated with eczema. Our results also suggest that in children at high-risk for atopic disease, a diverse adult-type microbiota in too early childhood may be a potential risk factor and further strengthen the importance of early microbiota characterization and potential dietary modification.

## Abbreviations

et rel.: And relatives; HITChip: Human intestinal tract chip; MCPP: Monte carlo permutation procedure; qPCR: Quantitative real-time polymerase chain reaction; rRNA: Ribosomal RNA.

## Competing interests

The authors declare that they have no competing interests.

## Authors’ contributions

MK and ES designed the original intervention study and organized the sample collection. Infants were clinically examined by MK. LN, WMdV, RS and SS designed the current study. LN performed faecal microbial DNA extraction, qPCR analyses and HITChip experiments. MR-S was involved in HITChip experiments. JN performed bioinformatic analyses. LN, RS and WMdV interpreted the results and wrote the paper. All authors read and approved the final manuscript.

## Supplementary Material

Additional file 1 Basic characteristics of the study subjects. Click here for file

Additional file 2 **Primers targeting *Bifidobacterium *genus and species used in this study.** Click here for file

Additional file 3 Differences in bifidobacterial composition of all children at 6 and 18 months of age as assessed by using quantitative PCR. Click here for file

Additional file 4 **The histograms showing the distribution of p-values obtained from the statistical analyses of species-like level of HITChip data at 18 months.** Each bar represents how many species-like groups gave a p-value in the given range when the effect of different factors on microbiota composition were analysed. Click here for file

Additional file 5 The microbiota differences of healthy and eczematous children from placebo group as assessed by HITChip analysis. Click here for file

Additional file 6 Bifidobacterial sub-communities in infants with eczema and healthy controls as assessed by quantitative PCR and HITChip analyses. Click here for file

Additional file 7 **Phylum-like (level 1) and genus-like (level 2) HITChip data used in this study.** Data is presented as log-transformed values. A letter A refers to 6 months samples and a letter D to 18 months samples, respectively. Click here for file

Additional file 8 **P-values obtained from the statistical analysis of phylum-like and genus-like groups of HITChip data at 18 months.** P-values are not corrected and therefore indicate trend-like differences in the abundance of individual bacterial groups between the groups of infants. Microbial groups that were over the detection level were included in the analysis. Click here for file

Additional file 9 The microbiota differences between the intervention groups (LGG or placebo) at the age of 18 months as assessed by HITChip analysis. Click here for file
