# Supplementary material for: Microarray analysis reveals marked intestinal microbiota aberrancy in infants having eczema compared to healthy children in at-risk for atopic disease
Source: BMC Microbiol. 2013 Jan 23;13:12. doi: 10.1186/1471-2180-13-12 (PMC3563445; doi:10.1186/1471-2180-13-12)
Supplement: Additional file 1 — Basic characteristics of the study subjects. [file 1471-2180-13-12-S1.pdf]

Additional file 1. Basic characteristics of the study subjects.

Subject	6mo sample	18mo sample	Intervention group	Total breastfeeding (months)	Health status (h/e)	First eczema symptoms (age in months)	SPT+-6kk	SPT+-24kk
**1**	yes	no	placebo	14.0	h	-	no	no
**2**	yes	yes	LGG	6.5	e	2.0	yes	yes
**3**	yes	yes	LGG	10.0	e	5.0	yes	yes
**4**	yes	yes	LGG	7.0	h	-	no	no
**5**	yes	yes	LGG	7.5	e	14.0	no	no
**6**	yes	yes	placebo	8.0	e	6.0	no	no
**7**	yes	yes	placebo	7.0	e	2.0	no	no
**8**	no	yes	LGG	11.0	h	-	no	no
**9**	yes	yes	LGG	7.0	e	5.0	no	yes
**10**	yes	yes	placebo	4.0	e	3.5	yes	yes
**11**	yes	yes	LGG	9.5	h	-	no	no
**12**	yes	no	placebo	19.0	h	-	no	no
**13**	yes	yes	placebo	10.0	h	-	no	no
**14**	no	yes	placebo	11.0	h	-	no	no
**15**	yes	no	LGG	12.0	h	-	no	no
**16**	yes	no	placebo	13.0	e	4.0	no	no
**17**	yes	yes	LGG	4.0	h	-	no	no
**18**	yes	yes	placebo	5.0	h	-	no	no
**19**	yes	no	placebo	11.0	e	7.0	no	NA
**20**	yes	no	placebo	6.0	h	-	no	no
**21**	yes	yes	placebo	11.0	e	16.0	no	no
**22**	yes	yes	placebo	12.0	e	3.0	no	no
**23**	yes	yes	LGG	11.0	h	-	no	no
**24**	yes	no	LGG	7.0	h	-	no	no
**25**	yes	yes	LGG	11.5	h	-	no	no
**26**	yes	no	LGG	4.0	h	-	no	no
**27**	no	yes	placebo	6.0	h	-	no	no
**28**	yes	no	placebo	7.0	h	-	no	no
**29**	yes	yes	placebo	10.0	h	-	no	no
**30**	yes	no	placebo	11.0	e	12.0	no	no
**31**	yes	yes	LGG	7.0	e	5.0	no	yes
**32**	yes	yes	placebo	9.0	e	2.0	no	no
**33**	yes	yes	placebo	6.0	h	-	no	NA
**34**	yes	yes	LGG	7.5	e	6.0	no	no

Health status: h = healthy, e = eczema
LGG indicates receiving supplementation with *L. rhamnosus* GG.
SPT+ = skin prick test positive; NA= information not available
