# Supplementary material for: Microarray analysis reveals marked intestinal microbiota aberrancy in infants having eczema compared to healthy children in at-risk for atopic disease
Source: BMC Microbiol. 2013 Jan 23;13:12. doi: 10.1186/1471-2180-13-12 (PMC3563445; doi:10.1186/1471-2180-13-12)
Supplement: Additional file 2 — Primers targeting Bifidobacterium genus and species used in this study. [file 1471-2180-13-12-S2.pdf]

Additional file 2. Primers targeting *Bifidobacterium* genus and species used in this study

**Target group/species**	**Forward primer (5' to 3')**	**Reverse primer (5' to 3')**	**Reference**
B. genus	GATTCTGGCTCAGGATGAACGC	CTGATAGGACGCGACCCCAT	(69,70)
B. adolescentis	GGATCGGCTGGAGCTTGCTCCG	CCCCGAAGGCTTGCTCCCAGT	(35)
B. bifidum	TGACCGACCTGCCCCATGCT	CCCATCCCACGCCGATAGAAT	(35)
B. breve	AATGCCGGATGCTCCATCACAC	GCCTTGCTCCCTAACAAAAGAGG	(35)
B. catenulatum group	GCCGGATGCTCCGACTCCT	ACCCGAAGGCTTGCTCCCGAT	(35)
B. longum group	TTCCAGTTGATCGCATGGTCTTCT	GGCTACCCGTCGAAGCCACG	(35)
