# Supplementary material for: Microarray analysis reveals marked intestinal microbiota aberrancy in infants having eczema compared to healthy children in at-risk for atopic disease
Source: BMC Microbiol. 2013 Jan 23;13:12. doi: 10.1186/1471-2180-13-12 (PMC3563445; doi:10.1186/1471-2180-13-12)
Supplement: Additional file 3 — Differences in bifidobacterial composition of all children at 6 and 18 months of age as assessed by using quantitative PCR. [file 1471-2180-13-12-S3.pdf]

Additional file 3. Differences in bifidobacterial composition of all children as assessed by using quantitative PCR.

**Bacterial group/species**	**Mean cell count (log)/g of faeces (SD)**		
	**6 months (n=31)**	**18 months (n=24)**	**p-value**
Bifidobacterium genus	10.48 (0.54)	10.03 (0.51)	< .001
B. longum group	10.00 (1.25)	9.01 (1.71)	0.01
B. adolescentis	6.61 (2.61)	5.25 (2.57)	0.05
B. bifidum	6.85 (2.60)	5.52 (2.23)	0.01
B. breve	7.91 (1.77)	6.31 (2.37)	< .001
B. catenulatum group	6.60 (2.21)	5.46 (2.44)	0.07
