# Supplementary figures and images for: Microarray analysis reveals marked intestinal microbiota aberrancy in infants having eczema compared to healthy children in at-risk for atopic disease
Source: BMC Microbiol. 2013 Jan 23;13:12. doi: 10.1186/1471-2180-13-12 (PMC3563445; doi:10.1186/1471-2180-13-12)

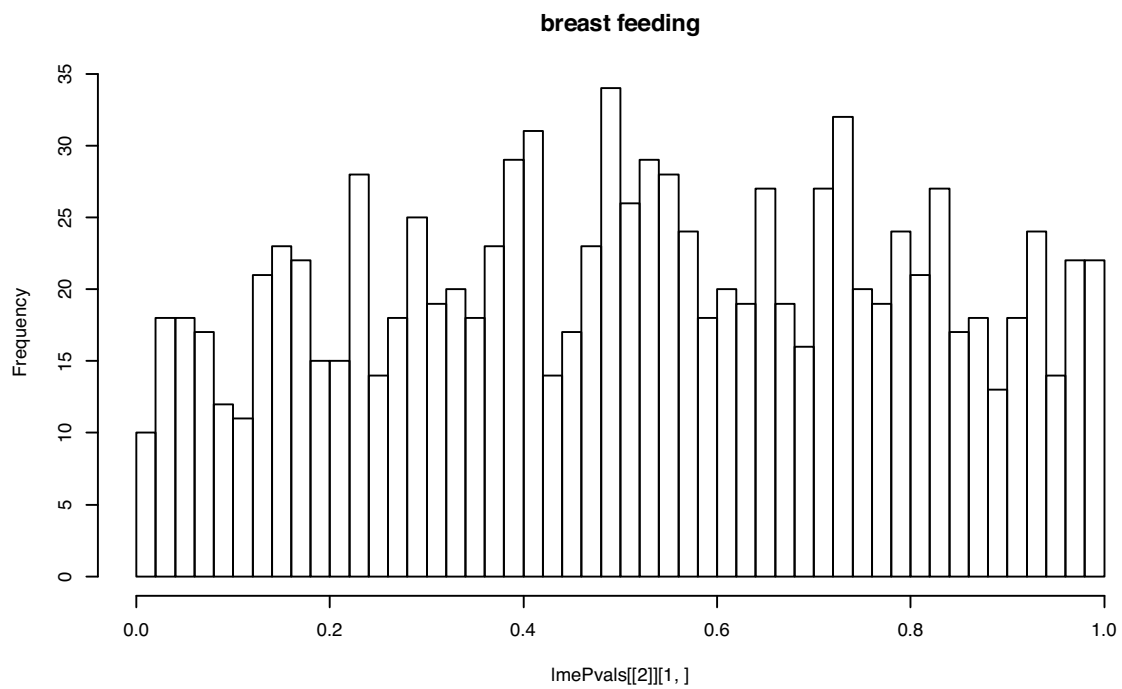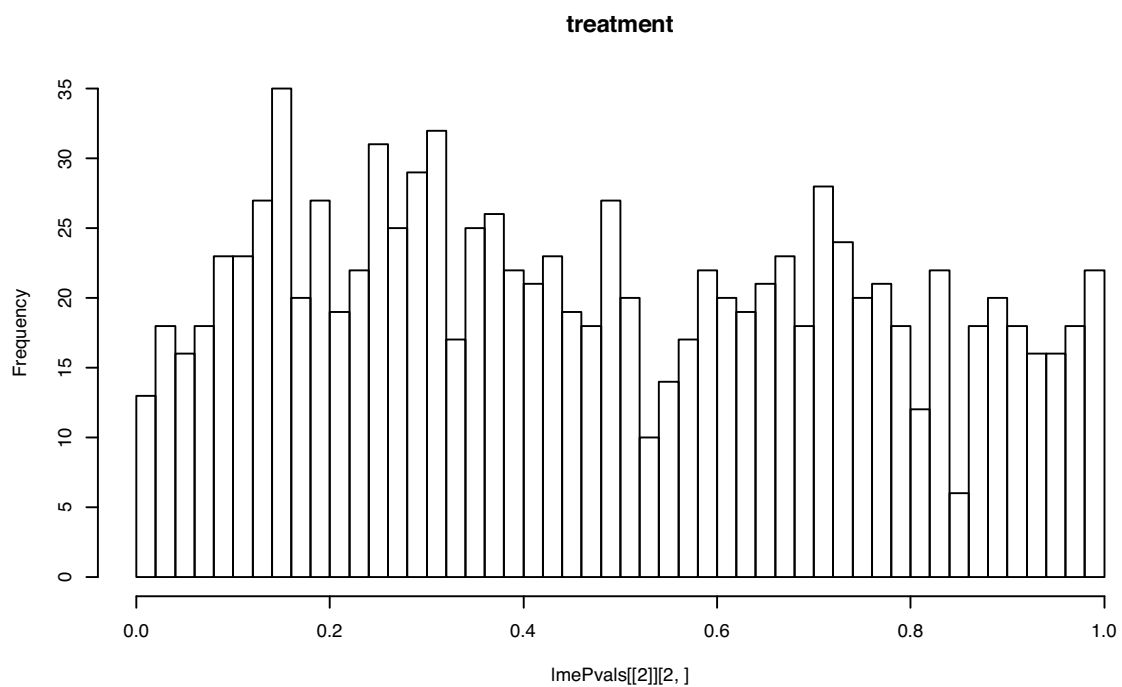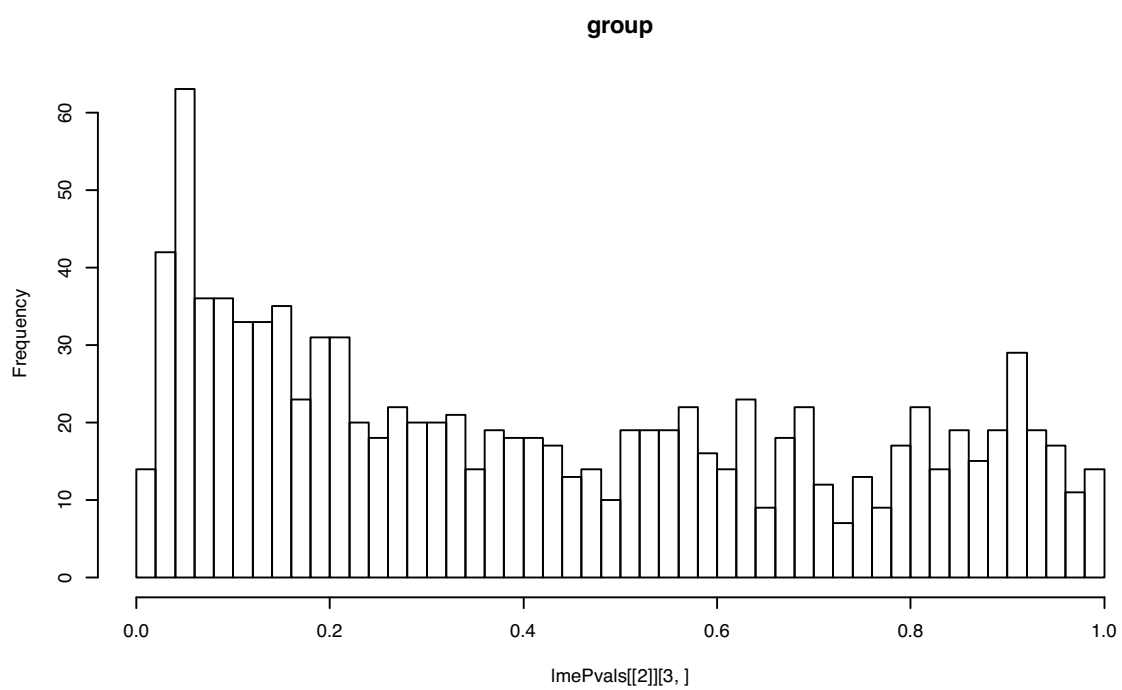

Supplement: Additional file 4 — The histograms showing the distribution of p-values obtained from the statistical analyses of species-like level of HITChip data at 18 months. Each bar represents how many species-like groups gave a p-value in the given range when the effect of different factors on microbiota composition were analysed. [file 1471-2180-13-12-S4.pdf]
