# Supplementary material for: Microarray analysis reveals marked intestinal microbiota aberrancy in infants having eczema compared to healthy children in at-risk for atopic disease
Source: BMC Microbiol. 2013 Jan 23;13:12. doi: 10.1186/1471-2180-13-12 (PMC3563445; doi:10.1186/1471-2180-13-12)
Supplement: Additional file 5 — The microbiota differences of healthy and eczematous children from placebo group as assessed by HITChip analysis. [file 1471-2180-13-12-S5.pdf]

Additional file 5. The microbiota differences of healthy and eczematous children from placebo group at the age of 18 months as assessed by HITChip analysis.

**Phylum/order**	**Genus-like phylogenetic group**	**Mean relative abundance* (SD)**		
		**18 months, placebo group**		**p-value**
		**Healthy (n=5)**	**Eczema (n=6)**	
Bacteroidetes		4.92 (5.70)	1.34 (0.25)	0.15
	B. fragilis et rel.	1.57 (2.15)	0.12 (0.02)	0.13
	B. ovatus et rel.	0.37 (0.62)	0.09 (0.02)	0.29
	B. plebeius et rel.	0.08 (0.03)	0.06 (0.01)	0.17
	B. stercoris et rel.	0.08 (0.03)	0.05 (0.01)	0.16
	B. uniformis et rel.	0.45 (0.59)	0.03 (0.01)	0.11
	B. vulgatus et rel.	1.29 (2.28)	0.18 (0.09)	0.26
	P. tannerae et rel.	0.06 (0.03)	0.04 (0.01)	0.22
Clostridium cluster IV	*C. leptum et rel.*	1.24 (1.98)	1.89 (1.10)	0.51
	R. bromii et rel.	0.33 (0.64)	0.57 (0.22)	0.41
	C. cellulosi et rel.	0.88 (1.15)	1.40 (0.70)	0.38
Clostridium cluster XIVa	*R. lactaris et rel.*	0.06 (0.03)	3.26 (3.71)	0.09
	C. nexile et rel.	1.60 (1.00)	1.62 (0.69)	0.96

* % of total HITChip signal
