# Supplementary material for: Microarray analysis reveals marked intestinal microbiota aberrancy in infants having eczema compared to healthy children in at-risk for atopic disease
Source: BMC Microbiol. 2013 Jan 23;13:12. doi: 10.1186/1471-2180-13-12 (PMC3563445; doi:10.1186/1471-2180-13-12)
Supplement: Additional file 6 — Bifidobacterial sub-communities in infants with eczema and healthy controls as assessed by quantitative PCR and HITChip analyses. [file 1471-2180-13-12-S6.pdf]

Additional file 6. Bifidobacterial sub-communities in infants with eczema and healthy controls as assessed by quantitative PCR and HITChip analyses.

**Bacterial group/species**	**Mean cell count (log)/g of faeces (SD)**					
	**6 months**		**p-value**	**18 months**		**p-value**
	**Healthy**	**Eczema**		**Healthy**	**Eczema**	
Bifidobacterium genus	10.62 (0.42)	10.35 (0.63)	0.111	10.15 (0.56)	9.91 (0.45)	0.26
B. longum group	10.32 (0.89)	9.65 (1.49)	0.120	9.41 (1.62)	8.61 (1.76)	0.26
B. adolescentis	6.66 (3.14)	6.56 (2.00)	0.913	5.65 (3.02)	4.81 (2.02)	0.43
B. bifidum	7.03 (2.67)	6.65 (2.60)	0.675	5.29 (2.34)	5.02 (2.22)	0.77
B. breve	7.80 (1.70)	8.03 (1.89)	0.707	5.52 (2.00)	7.04 (2.40)	0.13
B. catenulatum group	6.11 (2.22)	7.13 (2.15)	0.190	5.20 (2.32)	5.73 (2.64)	0.60
Bifidobacterium et rel. (HITChip)*	24.77 (16.74)	20.82 (15.31)	0.395	14.00 (11.49)	11.45 (7.85)	0.30

*Mean relative abundance (% of total HITChip signal) (SD)
