# Supplementary material for: Microarray analysis reveals marked intestinal microbiota aberrancy in infants having eczema compared to healthy children in at-risk for atopic disease
Source: BMC Microbiol. 2013 Jan 23;13:12. doi: 10.1186/1471-2180-13-12 (PMC3563445; doi:10.1186/1471-2180-13-12)
Supplement: Additional file 9 — The microbiota differences between the intervention groups (LGG or placebo) at the age of 18 months as assessed by HITChip analysis. [file 1471-2180-13-12-S9.pdf]

Additional file 9. The microbiota differences between the intervention groups (LGG or placebo) at the age of 18 months as assessed by HITChip analysis.

Genus-like phylogenetic group	Mean relative abundance* (SD)		
	**LGG (n=)**	**Placebo (n=)**	**p-value**
Anaerostipes caccae et rel.	2.89 (2.13)	1.18 (0.91)	0.03
Clostridium difficile et rel.	0.78 (0.60)	1.19 (0.85)	0.047
Eubacterium ventriosum et rel.	0.17 (0.11)	0.11 (0.07)	0.04

* % of total HITChip signal
